# *Helicobacter pylori* resistance in Hainan Province, China: investigating phenotypes and genotypes through whole-genome sequencing

**DOI:** 10.3389/fcimb.2024.1505166

**Published:** 2024-12-17

**Authors:** Yan-Ting Lv, Da Li, Da-Ya Zhang, Shi-Ju Chen, Run-Xiang Chen, Yang Wang, Wei-Zhong Yang, Lei Gao, Jun-Tao Zeng, Jian-Xin Xiong, Qiu-Ya Huang, Jing Huang, Qiao-Guan Zhang, Jia-Jia Chen, Fei-Hu Bai

**Affiliations:** ^1^ The Second School of Clinical Medicine, Hainan Medical University, Haikou, China; ^2^ Department of Digestive Endoscopy, The Second Affiliated Hospital of Hainan Medical University, Haikou, China; ^3^ Department of Gastroenterology, Sanya Central Hospital, Sanya, China; ^4^ Department of Gastroenterology, Hainan Second People’s Hospital, Wuzhishan, China; ^5^ Department of Gastroenterology, Dongfang People’s Hospital, Dongfang, China; ^6^ Department of Gastroenterology, Qionghai People’s Hospital, Qionghai, China; ^7^ Department of Gastroenterology, The Second Affiliated Hospital of Hainan Medical University, Haikou, China; ^8^ The Gastroenterology Clinical Medical Center of Hainan Province, Haikou, China

**Keywords:** *Helicobacter pylori*, antibiotic resistance, genotypic, phenotypic, lineage, whole-genome sequencing

## Abstract

*Helicobacter pylori* is increasingly resistant to antibiotics, significantly lowering eradication rates and posing a major public health challenge. This study investigated the distribution of antibiotic-resistant phenotypes and genotypes of *H. pylori* in Hainan Province. It determined the minimum inhibitory concentrations (MICs) of six antibiotics using the E-test method and detected resistance genes via Sanger sequencing. Furthermore, we compared resistance detection based on phenotypic analysis and whole genome sequencing (WGS) across 19 clinical isolates of *H. pylori*. A total of 140 *H. pylori* strains were isolated. The resistance rates to levofloxacin (LEV), clarithromycin (CLA), and metronidazole (MTZ) were 37.9%, 40.0%, and 93.6%, respectively. Notably, only 3.3% of the strains were susceptible to all six antibiotics. Multidrug-resistant strains accounted for 25.0% of the total, with no resistance detected to amoxicillin (AMX), tetracycline (TET), or furazolidone (FR) during the study period. Genotypic resistance to CLA and LEV showed near-perfect concordance with phenotypic resistance, with Kappa values of 0.910 and 0.938, respectively. Although all isolates were phenotypically sensitive to TET, 16 exhibited a mutation in the 16S rRNA gene (A926G). All strains harboring the R16H/C mutation and truncated *rdxA* were resistant to metronidazole, demonstrating a specificity of 100%. Therefore, FR, AMX, and TET are recommended as suitable empirical treatment options for *H. pylori* infections in this region. Genotypic analysis provides a reliable method for predicting resistance to CLA and LEV. WGS proves to be a valuable tool for identifying novel resistance loci in *H. pylori* and contributes to the phylogenetic classification of strains.

## Introduction

*Helicobacter pylori* is a gram-negative bacterium that resides on the luminal surface of the gastric epithelium, first identified by Warren and Marshall in 1983 (McColl, 2010). Since its discovery, *H. pylori* has emerged as one of the most prevalent pathogens worldwide, infecting more than half of the global population (Crowe, 2019). In China, the infection rate of *H. pylori* is approximately 44.2% (Chen et al., 2023). This bacterium has been established as the primary causative agent of various gastrointestinal diseases, including chronic gastritis, peptic ulcers (10-20%), gastric adenocarcinoma (1-2%), and gastric lymphoma (<1%) (Vakil and Megraud, 2007; Fukase et al., 2008; Bauer and Meyer, 2011).

The rise of antibiotic resistance has significantly compromised the eradication of *H. pylori*, with the success rate of the standard triple therapy declining from over 90% to less than 60% (Wang et al., 2014; Chen et al., 2023). Factors such as patient non-adherence to prescribed antibiotic regimens and the overuse of antibiotics in both healthcare and agricultural settings have exacerbated the spread of bacterial resistance (Sukri et al., 2022). Of particular concern is the resistance to clarithromycin (CLA), which severely undermines the effectiveness of triple therapy for *H. pylori* eradication. In response to this growing challenge, the World Health Organization (WHO) has identified CLA-resistant *H. pylori* as a critical priority for the development of new antibiotics (Tacconelli et al., 2018; Hanafiah et al., 2024).

In response to the growing challenge of antibiotic resistance, recent guidelines recommend bismuth-based quadruple therapy as the first-line empirical treatment for *H. pylori* eradication in our country (Zhou et al., 2022). Additionally, recent studies have shown that vonoprazan-based regimens yield favorable outcomes in patients infected with *H. pylori*. The vonoprazan-amoxicillin (VA) regimen achieves an eradication rate of 82.8%, the vonoprazan-amoxicillin-clarithromycin (VAC) combination reaches 89.1%, and the bismuth-containing quadruple therapy with vonoprazan (VBQT) attains a 91.8% eradication rate. Importantly, increasing the frequency of amoxicillin (AMX) administration and extending the treatment duration further enhance the efficacy of these regimens (Okubo et al., 2020; Qian et al., 2023; Liu et al., 2024). Moreover, there are notable differences in infection prevalence across different geographical regions within a country and between countries, as well as variations in antimicrobial resistance rates (Hooi et al., 2017).

Personalized therapy, guided by antibiotic resistance profiling—including both phenotypic and genotypic assessments—represents a critical strategy to address eradication failure (Xiong et al., 2023). Genotypic resistance testing for *H. pylori* has emerged as a promising alternative to traditional phenotypic resistance testing, overcoming the challenges of stringent culture requirements and low cultivation success rates. Whole-genome sequencing (WGS) has recently proven to be a powerful tool for identifying novel antibiotic resistance loci and serves as a complementary method to conventional antibiotic susceptibility testing (Fauzia et al., 2023). In this study, we applied WGS to 19 clinical isolates of *H. pylori* to elucidate the local strain lineages and compared the genotypic and phenotypic resistance profiles of these strains against six antibiotics, identifying novel resistance mutation sites. Additionally, WGS was utilized to characterize the strain lineages.

## Materials and methods

### Study subjects

**Figure 1:**
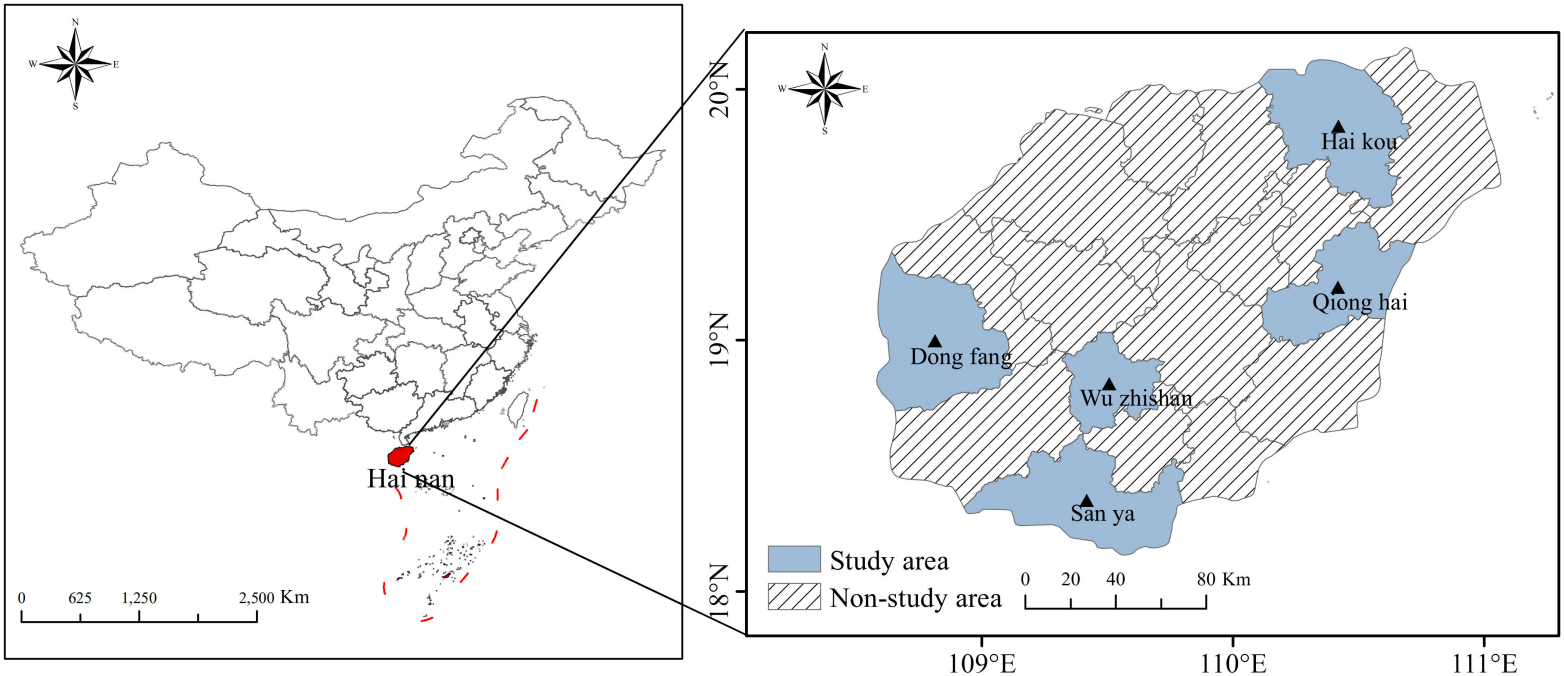
Map of Hainan Province and the distribution of samples. Samples were collected from five regions, including hospitals in Haikou, Sanya, Qionghai, Wuzhishan, and Dongfang.

From May to September 2023, a total of 193 patients with *H. pylori* infection were evaluated across five hospitals in Hainan (Haikou, Sanya, Qionghai, Wuzhishan, and Dongfang) due to symptoms of indigestion (**Figure 1**). A stratified sampling survey was conducted among hospital visitors. The inclusion criteria were patients confirmed to have *H. pylori* infection through a rapid urease test or urea breath test (UBT), who also provided informed consent to participate in the study. The exclusion criteria were as follows: (1) use of proton pump inhibitors (PPI), gastric mucosal protectants, AMX, CLA, metronidazole, or sulfonamide antibiotics within the past month; (2) severe mental illnesses, including anxiety, depression, or cognitive impairments; (3) pregnancy or breastfeeding; and (4) age under 18 years.

Gastrointestinal diseases were diagnosed through gastroscopy and histopathological examination. During upper gastrointestinal endoscopy, gastric mucosal biopsies were taken from the greater curvature of the gastric body and antrum. This study was conducted in accordance with the Declaration of Helsinki (revised in 2013) and the Maastricht Consensus Report (latest revision in 2022). Approval for the study was obtained from the Institutional Review Board of the Second Affiliated Hospital of Hainan Medical University (approval number: LW202149), and written informed consent was obtained from all participants.

### Cultivation and identification of *H. pylori*

*H. pylori* strains were isolated from patients diagnosed with various gastric diseases during routine endoscopic examinations and biopsies. The strains were cultured on Columbia agar plates supplemented with 5% defibrinated sheep blood under microaerophilic conditions (5% O_2_, 10% CO_2_, and 85% N_2_) for approximately 3-4 days. The identification of *H. pylori* was confirmed by assessing colony morphology, Gram staining, and positive results from oxidase, catalase, and urease tests. All isolates were subsequently preserved at -80°C in sterile brain heart infusion (BHI) broth containing 20% glycerol for further analysis.

### Antibiotic susceptibility testing

The antibiotic susceptibility of the *H. pylori* strains was initially screened using the Kirby-Bauer disk diffusion method to assess basic drug responses. Further sensitivity testing was performed using the Etest method (BIO-KONT, Wenzhou, China) for metronidazole, CLA, levofloxacin (LEV), AMX, tetracycline (TET), and furazolidone (FR). The reference strain *H. pylori* 26695 was used as a quality control standard. Pure colonies were resuspended in 750 mL of sterile saline to match the turbidity of the McFarland 3 standard. This suspension was uniformly spread onto Columbia agar plates containing 5% sheep blood using a disposable sterile cotton swab. After allowing the plates to dry naturally, antibiotic susceptibility test strips were applied vertically. The plates were then incubated under microaerophilic conditions at 37°C for 72 hours, after which the minimum inhibitory concentrations (MICs) were determined. Resistance breakpoints were defined according to the EUCAST (version 11.0) guidelines as follows: CLA (>0.5 μg/mL), LEV (>1 μg/mL), TET (>1 μg/mL), metronidazole (>8 μg/mL), and AMX (>0.125 μg/mL) (Fazal, 2019). For FR, in the absence of an established standard resistance breakpoint, a threshold of >4 μg/mL was adopted based on prior research (Goh and Navaratnam, 2011).

### Detection of antibiotic resistance-related genetic variants

Comprehensive sequence alignment analyses were performed using the reference strain genome *H. pylori* 26695 (NC_000915.1) to identify genetic variants and mutations associated with resistance to various antibiotics (Tshibangu-Kabamba and Yamaoka, 2021). The analysis focused on key resistance mechanisms, including mutations in the *rdxA* gene linked to metronidazole resistance, 23S rRNA for CLA resistance, the *gyrA* gene for LEV resistance, and the *pbp1* genes for AMX resistance. Additionally, mutations in 16S rRNA were investigated for TET resistance, and mutations in the *oorD* and *porD* genes were examined for FR resistance. The study also explored the correlations between phenotypic resistance and genotypic characteristics, as well as the identification of novel mutations in strains that exhibited phenotypic resistance. Furthermore, the presence of multidrug resistance (MDR), defined as resistance to three or more antibiotic classes (Rokkas and Ekmektzoglou, 2023), was investigated to identify mutations associated with this significant global health concern.

### Genomic analysis of *H. pylori* isolates

Clinical isolates of *H. pylori* were cultured on blood agar to facilitate genomic DNA extraction. Following incubation under microaerophilic conditions at 37°C for 3-4 days, bacterial colonies were collected with a sterile loop and resuspended in saline solution. Genomic DNA was extracted using the HiPure Bacterial DNA Kit (Magen Inc.) according to the manufacturer’s instructions. The integrity and purity of the extracted DNA were assessed using a NanoDrop One spectrophotometer (Thermo Fisher Scientific, Inc.). For whole-genome sequencing (WGS), library preparation was carried out utilizing the KAPA HyperPlus Kit (Illumina) in a batch of 96 reactions, adhering to established protocols. Sequencing procedures were executed on the Illumina MiSeq platform (Illumina, Inc.). Sequence quality assessment was performed with FastQC software (v0.11.7), and adapter sequences were removed, followed by trimming using Fastp software (v0.12.5). The resultant sequence reads were subsequently assembled with SPAdes software (version 3.13.0). The coverage for the sequencing spanned from 168x to 347x, averaging 287x. Comprehensive quality metrics for the genomes sequenced, such as coverage, genome length, and the N50, N75, L50 and L75 values, can be found in **Supplementary Table S1**.

### Phylogenetic tree construction

To identify resistance mutations, we selected 61 *H. pylori* genomes from various regions as reference sequences. Using MEGA software (v10.0.5) and Orthofinder (Kumar et al., 2018; Gutiérrez et al., 2024), we extracted 161 sets of conserved single-copy genes from a total of 80 genomes, including 19 strains from this study. Multiple sequence alignments were performed with MAFFT, followed by the construction of a phylogenetic tree using PhyML software based on the maximum likelihood method. This approach allowed us to determine the clustering of the 19 clinical strain genomes from our samples in comparison to previously defined *H. pylori* sequence types. The sequence setting parameters for these conserved single-copy genes, used for constructing the phylogenetic tree, are detailed in **Supplementary Table S4**. The genomes analyzed include the 19 sequenced in this study, along with 61 others previously reported in the literature.

### Statistical analysis

The concordance between antibiotic resistance phenotypes and genotypes was quantified using the Kappa statistic. Kappa values were interpreted as follows: <0.2 indicating slight agreement, 0.21–0.4 fair agreement, 0.41–0.6 moderate agreement, 0.61–0.8 good agreement, and >0.8 almost perfect agreement. The association between single nucleotide mutation and antibiotic resistance was evaluated employing either Chi-square tests or Fisher’s exact tests. Statistical significance for all analyses was determined with a p-value threshold of less than 0.05. All computations and visualizations were conducted using R software (version 3.6.4).

### Data availability

In this study, the genomes of 19 *H. pylori* strains, each isolated from distinct clinical samples, were sequenced, assembled, and annotated. These genomes have been submitted to the NCBI BioProject database, registered under the accession number PRJNA1171892.

## Results

### Patient characteristics

Antibiotic susceptibility testing was conducted on 193 participants, from which 140 *H. pylori* strains were successfully isolated. This resulted in a 73% success rate for isolation and culture in this study. **Table 1** presents the baseline characteristics of the included subjects: 57 males (40.7%) and 83 females (59.3%). The average age of the participants was 49.01 ± 13.06 years. The geographic distribution of the strains was as follows: 42 cases from Haikou City (30.0%), 20 cases from Qionghai City (14.2%), 32 cases from Dongfang City (22.8%), 25 cases from Sanya City (17.8%), and 21 cases from Wuzhishan City (15.0%).

**Table 1:** Basic information for patients with *H. pylori* isolated strains.

Group	Total
Total	140
Gender	
Female	83 (59.29%)
Male	57 (40.71%)
Mean age ± SD ((years)	49.15 ± 12.72
Range	
≤44 years	54 (38.57%)
45-59 years	51 (36.43%)
≥60 years	35 (25.00%)
Eradication history	
None	107 (76.43%)
Yes	33 (23.57%)
Endoscopic findings	
Gastric/duodenal erosions	44 (31.43%)
Bile reflux gastritis	10 (7.14%)
Peptic ulcer diseases	27 (19.29%)
Gastric polyps	9 (6.43%)
Others	50 (35.71%)
Location	
Haikou	42 (30.00%)
Dongfang	32 (22.86%)
Sanya	25 (17.86%)
Wuzhishan	21 (15.00%)
Qionghai	20 (14.29%)

### Phenotypic antibiotic resistance of *H. pylori* isolates

The majority of the strains exhibited high resistance to MTZ, LEV, and CLA. The overall resistance rate for CLA was 40.0%, while the resistance rate for LEV was slightly lower at 37.9%, with similar patterns observed across all regions. Notably, Wuzhishan City showed relatively lower resistance rates for both CLA and LEV at 12.5%. The resistance rate for MTZ was alarmingly high at 93.6%, reaching 100% in certain areas. During the study period, no resistance was observed for FR, AMX, or TET.

**Figure 2:**
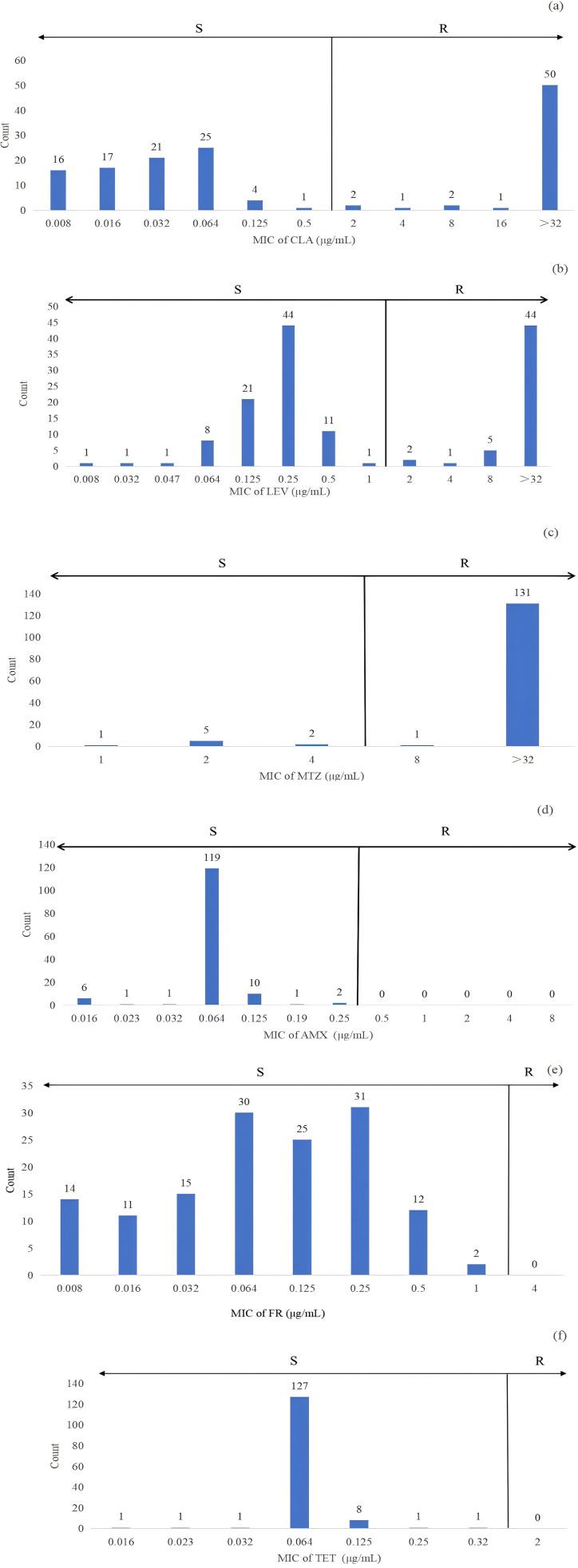
The distribution of MIC for strains sensitive and resistant to clarithromycin (CLA) **(A)**, levofloxacin (LEV) **(B)**, metronidazole (MTZ) **(C)**, amoxicillin (AMX) **(D)**, furazolidone (FR) **(E)** and tetracycline (TET) **(F)** was analyzed in 140 clinical *H*. *pylori* strains using the E-test. Antibiotic resistance was assessed based on the breakpoints defined in the European Committee on Antimicrobial Susceptibility Testing (EUCAST) guidelines. Specifically, resistance to CLA, LEV, TET, MTZ, and AMX was categorized according to these criteria. For FR, a resistance breakpoint of 4 μg/mL was used, as indicated in previous studies. In this context, ‘R’ indicates resistance, and ‘S’ indicates susceptibility.

Further analysis revealed that *H. pylori* resistance was associated with high MIC levels. Among MTZ-resistant strains, 92.1% (130/140) had MIC values exceeding 32 μg/mL, as depicted in **Figure 2**. For LEV-resistant strains, 31.2% (44/140) had MIC values over 32 μg/mL, while 35.4% (50/140) of CLA-resistant strains had MIC values above 32 μg/mL (**Figure 2**).

The 140 strains exhibited diverse antibiotic resistance patterns (**Table 2**), with 5.0% of the strains being sensitive to all six antibiotics tested. The most common single antibiotic resistance was MTZ, observed in 42.9% of the strains. Regarding multidrug-resistant strains, the majority exhibited dual or triple resistance. The most frequent dual resistance pattern was CLA+MTZ, found in 39.3% of the strains, while the rates of dual resistance for CLA+LEV and LEV+MTZ were 0.7% and 36.4%, respectively. A triple antibiotic resistance pattern involving CLA+LEV+MTZ was observed in 25.0% of the strains. No strains were found to be resistant to combinations of four or five antibiotics (**Table 2**). The rates of MDR varied by geographical area, with Haikou City and Wuzhishan City showing relatively lower rates of 14.3% and 19.0%, respectively, while Sanya City and Qionghai City exhibited higher rates of 36.0% and 45.0%. Review of medical records indicated that among these patients, 23.57% had primary infections and had not received eradication treatment.

**Table 2:** Distribution of resistance patterns among *H. pylori* strains.

Susceptibility test results	Value
Sensitive to all ABs	7 (5.00%)
Single	61 (43.57%)
CLA	56 (40.00%)
LEV	53 (37.86%)
MTZ	131 (93.57%)
AMX	0
TET	0
FR	0
Double	107 (76.43%)
MTZ+LEV	55 (39.29%)
MTZ + CLA	51 (36.43%)
LEV + CLA	1 (0.71%)
Triple	35 (25.00%)
MTZ + LEV + CLA	35 (25.00%)
MTZ + LEV + TET	0
MTZ + LEV + AMX	0
MTZ + CLA + AMX	0
Quadruple	0
Resistance to all ABs	0

AB, antibiotic.

**Figure 3:**
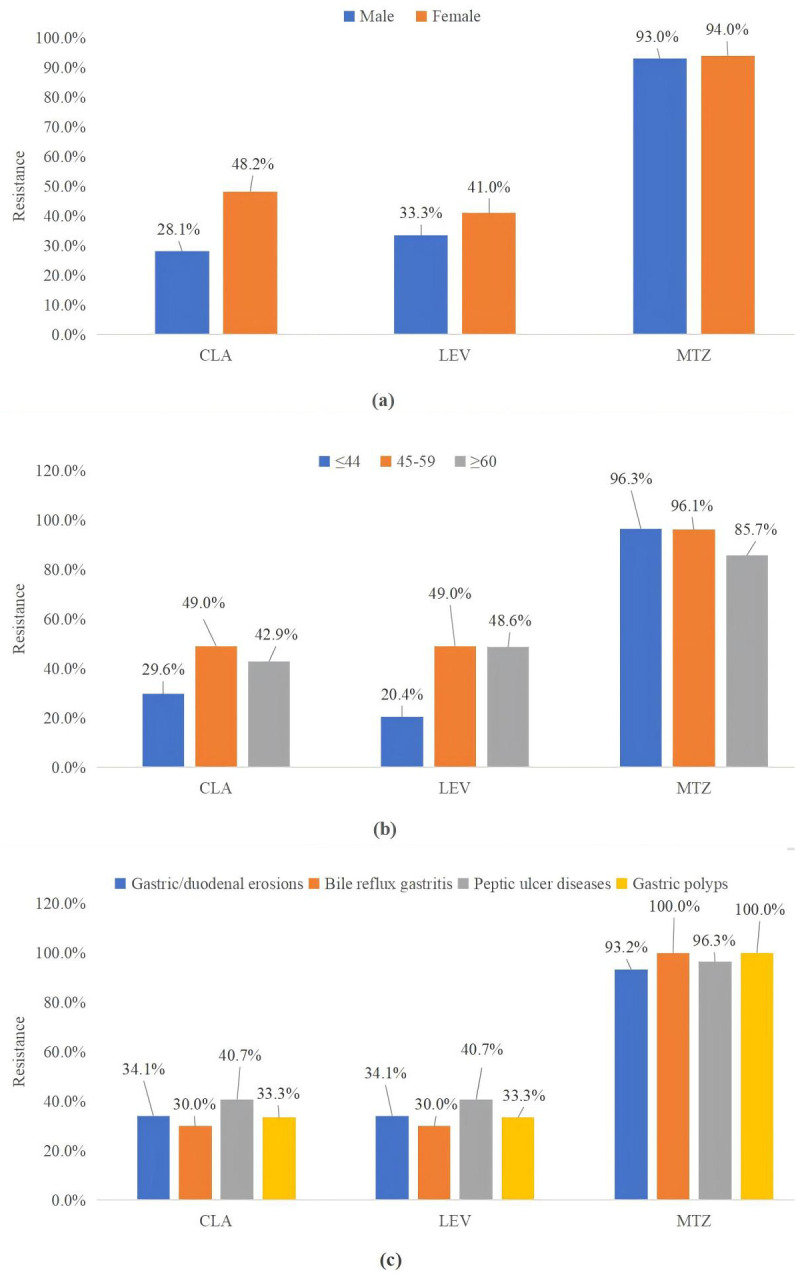
CLA, LEV, and MTZ resistance were classified by sex **(A)**, age **(B)**, and endoscopic findings **(C)**. MTZ, metronidazole; CLA, clarithromycin; LEV, levofloxacin.

Factors influencing resistance to CLA, LEV, and MTZ are illustrated in **Figure 3**. When stratified by gender, females exhibited significantly higher resistance rates to CLA compared to males (*p* = 0.017, *p* < 0.05). However, gender did not significantly correlate with resistance to LEV (*p* = 0.360) or MTZ (*p* = 0.814). Additionally, no significant differences in resistance rates to these three antibiotics were observed when participants were categorized by age or the presence or absence of ulcers.

### Genotypic determination of antibiotic resistance

To gain a deeper understanding of the molecular basis of antibiotic resistance, we not only assessed the efficacy of antibiotics against the bacteria but also conducted PCR analysis on biopsy samples to identify specific genotypes associated with antibiotic resistance (**Table 3**). Our analysis revealed a significant prevalence of resistant genotypes, particularly for CLA, MTZ, and LEV. Within these genotypes, several key mutation patterns were identified. For CLA resistance, the A2143G mutation was predominant, accounting for 35.7% of the cases. For LEV resistance, a comparative analysis between LEV-sensitive and LEV-resistant strains revealed significant mutational differences, with the N87K mutation being particularly notable, occurring at a frequency of 15.0%. Notably, no N87T mutations were detected in our dataset. Regarding AMX resistance, mutations in the pbp1A gene were observed in 22.9% of the strains, with the most common mutation being Thr593Ala, which was present in 7.9% of the cases. For TET resistance, potential mutations in the 16S rRNA gene, characterized by single or multiple base substitutions in AGA926_928 > GGA, were identified, with an overall occurrence rate of 7.1%.

**Table 3:** Correlation between point mutations and amino acid substitutions of antibiotic resistance genes and phenotypic resistance in *H. pylori*.

Mutation type	Percentage of Mutation, n (%)	Phenotype, n or n (%)		p value
		Resistant	Sensitive	
CLA (23S rRNA)	50/140 (35.71%)	50	0	
A2143G	50 (35.71%)	50 (100%)	0 (0)	0.000
LEV (*gyr*A)	49/140 (35.00%)	48	1	
N87K/I/Y	28 (57.14%)	28 (58.33%)	1 (100%)	0.296
D91N/G/Y	21 (42.86%)	21 (43.75%)	0 (0)	0.057
MTZ (rdxA)	140/140 (100%)	131	9	
R16H/C	24 (17.14%)	22 (16.79%)	2 (22.22%)	0.671
M21A	4 (2.86%)	3 (2.29%)	1 (11.11%)	0.576
T31E	74 (52.86%)	70 (53.4%)	4 (44.44%)	1.000
Y47C	4 (2.86%)	4 (3.05%)	0 (0)	1.000
H53R	67 (47.86%)	63 (48.09%)	4 (44.44%)	0.633
D59N	72 (51.43%)	68 (51.91%)	4 (44.44%)	0.628
L62V	52 (37.14%)	48 (36.64%)	4 (44.44%)	0.565
A67V	2 (1.43%)	2 (1.53%)	0 (0)	1.000
A8OT	5 (3.57%)	5 (3.82%)	0 (0)	1.000
S88P	70 (50.00%)	66 (50.38%)	4 (44.44%)	0.623
G98S/N	70 (50.00%)	66 (50.38%)	4 (44.44%)	0.623
R131K	70 (50.00%)	66 (50.38%)	4 (44.44%)	0.623
V1721	70 (50.00%)	66 (50.38%)	4 (44.44%)	0.623
V204I	4 (2.86%)	4 (3.05%)	0 (0)	1.000
AMX (pbp1A)	32/140 (22.86%)	0	140	
Thr593Ala	11 (34.38%)	0 (0)	11 (7.86%)	1.000
Thr556Ser	7 (21.89%)	0 (0)	7 (5.00%)	1.000
Asn562Tyr	7 (21.89%)	0 (0)	7 (5.01%)	1.000
Phe366Leu	3 (9.38%)	0 (0)	3 (2.14%)	1.000
Val374Leu	3 (9.38%)	0 (0)	3 (2.14%)	1.000
Ser414Arg	1 (3.13%)	0 (0)	1 (0.71%)	1.000
TET (16S rRNA)	16/140 (11.43%)	0	140	
AGA926_928>AGC	1 (6.25%)	0 (0)	1 (0.71%)	1.000
AGA926_928>CGA	1 (6.25%)	0 (0)	1 (0.71%)	1.000
AGA926_928>GGA	10 (7.1%)	0 (0)	10 (7.1%)	1.000
AGA926_928>GGC	1 (6.25%)	0 (0)	1 (0.71%)	1.000
AGA926_928>TGA	3 (18.75%)	0 (0)	3 (2.14%)	1.000
FR (porD)	27/140 (19.29%)	0	140	
A356G	20 (74.07%)	0 (0)	20 (14.29%)	1.000
C357T	21 (77.77%)	0 (0)	21 (15.00%)	1.000
G353A	26 (96.30%)	0 (0)	26 (18.57%)	1.000
FR (oorD)	140/140 (100%)	0	140	1.000
A41G	140 (100%)	0 (0)	140 (100%)	1.000
A122G	136 (97.14%)	0 (0)	136 (97.14%)	1.000
A335G	140 (100%)	0 (0)	140 (100%)	1.000
C349A	92 (65.71%)	0 (0)	92 (65.71%)	1.000
C349G	50 (35.71%)	0 (0)	50 (35.71%)	1.000

MTZ, metronidazole; C, CLA, clarithromycin; A, AMX, amoxicillin; L, LEV, levofloxacin; T, TET, tetracycline; F, FR, furazolidone.

Resistance to MTZ is primarily attributed to mutations in the *rdxA* and *frxA* genes, which reduce the efficacy of reductase activity, leading to insufficient activation of MTZ (Tshibangu-Kabamba and Yamaoka, 2021). *RdxA* mutations are considered to be central to the potential mechanisms of resistance, although definitive conclusions are still lacking. This study cataloged all mutations in the rdxA gene linked to MTZ resistance, identifying R16H/C and M21A as the mutations most strongly correlated with MTZ resistance (Zhang et al., 2020). Among the 131 phenotypically resistant strains, 22 were found to carry R16H/C mutations. Additionally, 13 strains exhibited truncated *rdxA* genes due to nonsense mutations at positions 15, 35, 50, 75, 76, 110, 146, and 209 (**Supplementary Table S2**), leading to the production of truncated or non-functional proteins. Truncation of the *rdxA* gene was observed in only 11.5% of the resistant strains, with these isolates frequently displaying multiple nonsynonymous point mutations previously linked to MTZ resistance (Mannion et al., 2021). Furthermore, regardless of the resistance phenotype, several amino acid substitutions were identified in most strains, including T31E, H53R, D59N, L62V, S88P, G98S, R131K, and V172.

In this study, the occurrence rate of mutations at reported sites in *porD* associated with FR resistance was 19.3%, while the occurrence rate for *oorD* mutations associated with FR resistance was 100%, suggesting that this mutation may be a primary mechanism of FR resistance. Further research is needed to decipher the relationship between multiple resistance gene mutations and actual resistance phenotypes to better understand how these mutations contribute to enhanced antibiotic resistance. Among the antibiotics with high resistance rates, the resistance pattern for MTZ was particularly complex, indicating that resistance resulting from multiple mutational sites is more prevalent than resistance caused by a single mutation.

**Table 4** presents the alignment between phenotypic and genotypic resistance of *H. pylori* to six antibiotics was examined. Mutations at the 23S rRNA (A2143G) and *gyrA* (N87, D91) loci showed strong agreement in determining resistance to CLA (Kappa = 0.910, 95% CI, 0.838 to 0.980) and LEV (Kappa = 0.938, 95% CI, 0.879 to 0.998). Conversely, the correlation between phenotypic and genotypic resistance for AMX, FR, TET, and metronidazole did not reach statistical significance.

**Table 4:** Consistency of phenotypic resistance with genotypic resistance.

Antibiotic	Genotypic resistance or susceptibility	No. of phenotypically resistant or susceptible strains		Sensitivity (%)	Specificity (%)	Accuracy (%)	Kappa (95% CI)	P value by Fisher’s exact test	P value
		R	S						
CLA	R	50	0	93.33%	100.00%	95.71%	0.91 (0.838, 0.980)	0.000	0.000
	S	6	84						
LEV	R	49	0	95.60%	100.00%	97.14%	0.938 (0.879, 0.998)	0.000	0.000
	S	4	87						
AMX	R	0	25		82.14%	82.14%	0.000 (-0.339, 0.339)		0.791
	S	0	115						
FR	R	0	27		80.71%	80.71%	0.000 (-0.339, 0.339)		0.837
	S	0	113						
TET	R	0	16		88.57%	88.57%	0.000 (-0.461, 0.461)		0.543
	S	0	124						
MTZ	R	131	9	100.00%		93.57%	0.000 (-0.632, 0.632)		0.317
	S	0	0						

### Mutation analysis using WGS

**Figure 4:**
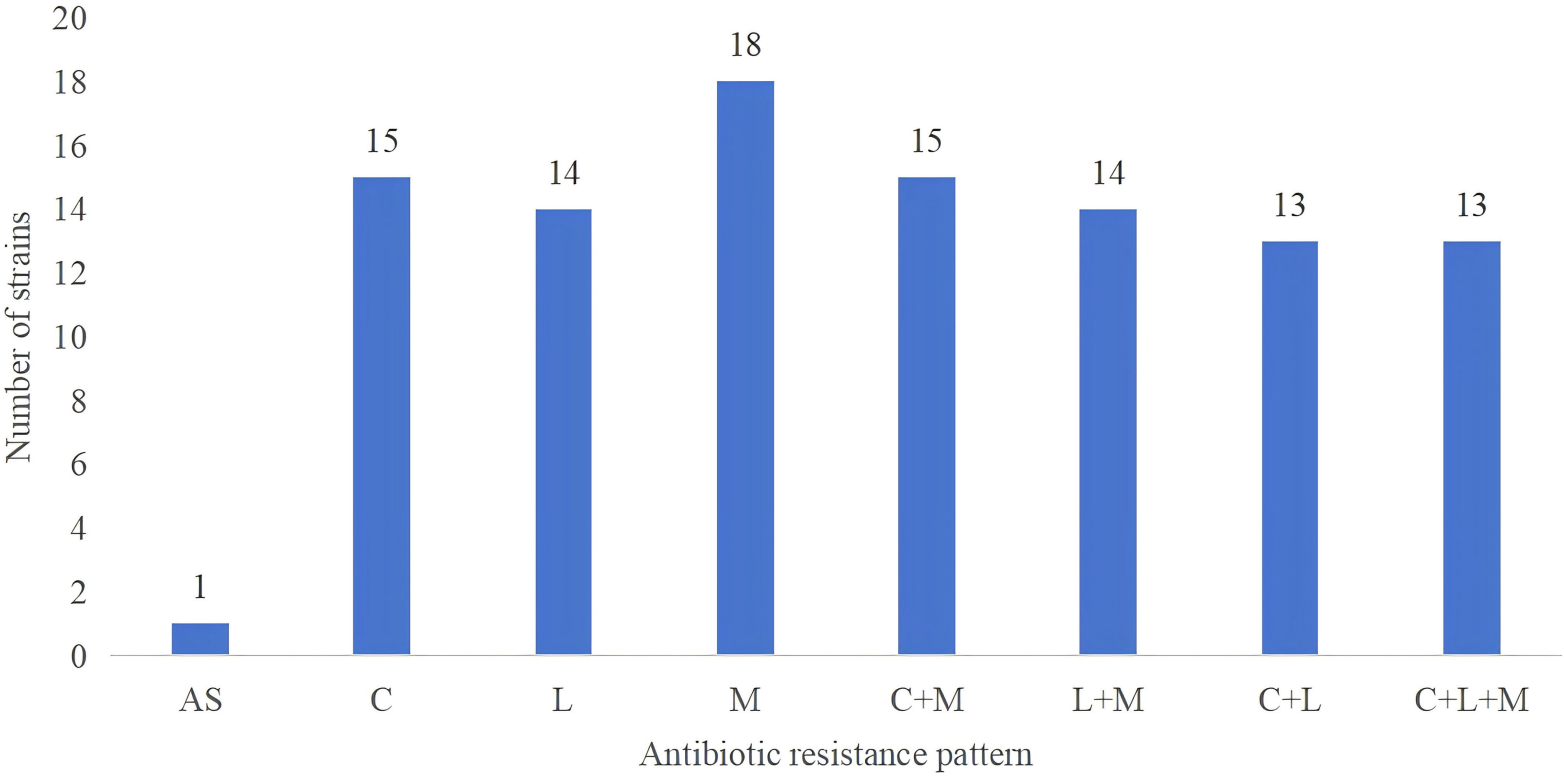
Distribution of antibiotic resistance patterns in strains based on whole genome sequencing. The study includes 19 clinical isolates of *H. pylori*, demonstrating diverse resistance patterns including susceptibility to all antibiotics, single-drug resistance, dual-drug resistance, and triple-drug resistance. ‘AS’ indicates susceptibility to all antibiotics; ‘M’ stands for Metronidazole (MTZ); ‘C’ represents Clarithromycin (CLA); ‘L’ denotes Levofloxacin (LEV).

1. WGS was performed on 19 of the successfully cultured 140 strains, including 13 MDR strains (**Table 5**). **Figure 4** summarizes the distribution of antibiotic resistance patterns in strains based on whole genome sequencing. Annotated genomes were analyzed to retrieve gene sequences linked to resistance, aiming to identify potential mutations conferring resistance. The study focused on mutations previously associated with resistance to AMX, TET, CLA, LEV, MTZ, and FR, and explored their correlation with phenotypic results. Among the 19 isolates, resistance to CLA and LEV was more prevalent in strains obtained from patients with a history of *H. pylori* infection (CLA: 57.89% vs. 10.53%, p=0.003; LEV: 57.89% vs. 15.79%, p=0.007). In contrast, mutations associated with resistance to AMX, TET, and FR were identified in the *pbp1A* (Rimbara et al., 2008; Gong and Yuan, 2018; Windham and Merrell, 2022), 16S rRNA (Malfertheiner et al., 2023), and *porD* genes of the isolates (Tshibangu-Kabamba and Yamaoka, 2021).

**Table 5:** The distribution of antibiotic resistance of *H. pylori* isolated strains by age, gender and infection status.

Characteristic	Total, n (%)	Antibiotic-resistant strain, n (%)					
		CLA	LEV	MTZ	AMX	FR	TET
No. (%) of patients ages (yr):							
18-35	2	2	2	2	0	0	0
35-50	7	5	3	7	0	0	0
50-75	10	8	9	9	0	0	0
P value		0.165	0.046	0.115			
No. (%) of patients by gender							
Female	11	9	9	11	0	0	0
Male	8	6	5	7	0	0	0
P value		0.439	0.285	0.346			
Ethnicity							
Li	4 (21.05)	2 (50.00)	2 (50.00)	4 (100)	0 (0.00)	0 (0.00)	0 (0.00)
Han	15 (78.95)	13 (86.67)	12 (80.00)	14 (93.33)	0 (0.00)	0 (0.00)	0 (0.00)
P value		0.000	0.000	0.002			
Endoscopic findings							
Gastric/duodenal erosions	5 (26.32)	4 (80.00)	3 (60.00)	5 (100)	0 (0.00)	0 (0.00)	0 (0.00)
Bile reflux gastritis	1 (5.26)	1 (100)	1 (100)	1 (100)	0 (0.00)	0 (0.00)	0 (0.00)
Peptic ulcer diseases	2 (10.53)	2 (100)	2 (100)	2 (100)	0 (0.00)	0 (0.00)	0 (0.00)
Gastric polyps	1 (5.26)	1 (100)	1 (100)	1 (100)	0 (0.00)	0 (0.00)	0 (0.00)
chronic gastritis	19 (100)	15 (78.95)	14 (73.64)	18 (94.73)	0 (0.00)	0 (0.00)	0 (0.00)
P value		0.000	0.000	0.000			
No. (%) of patients by infection status							
Primary	6 (31.58)	2 (33.33)	3 (50.00)	5 (83.33)	0 (0.00)	0 (0.00)	0 (0.00)
Secondary or tertiary	13 (68.42)	11 (69.23)	11 (69.23)	13 (100)	0 (0.00)	0 (0.00)	0 (0.00)
P value		0.003	0.007	0.018			

2. Genotypic determination of antimicrobial resistance.

The most common genetic mutation associated with CLA resistance is the point mutation in the 23S rRNA gene, specifically A2142G/C or A2143G (Lyu et al., 2023). Among the isolates, 15 demonstrated phenotypic resistance to CLA, all of which carried the A2143G mutation. The A2142G mutation was absent in all isolates. A kappa consistency analysis was conducted to assess the correlation between genotypic and phenotypic resistance using the A2143G mutation, yielding a kappa coefficient of 1.000 with a 95% CI of 1.0 to 1.0 (**Table 6**). This indicates perfect concordance between phenotypic and genotypic assessments in predicting CLA resistance in these isolates. Additionally, none of the isolates carried mutations such as A2142C, A2115G, A2144T, G2141A, C2147G, T2289C, or T2190C (Marques et al., 2020; Zhou et al., 2022), which have previously been associated with low-level resistance. Similarly, mutations like C2173T and G2212A, found only in resistant strains, were not observed in our isolates (Hansomburana et al., 2012; Zhao et al., 2014; Rosli et al., 2023).

**Table 6:** Phenotypic and genotypic resistance consistency analysis of H. pylori isolates based on WGS.

Antibiotic	Genotypic resistance or susceptibility	No. of phenotypically resistant or susceptible strains		Sensitivity (%)	Specificity (%)	Accuracy (%)	Kappa coefficient (95% CI)	P value by Fisher’s exact test
		R	S					
CLA	R	15	0	100.00%	100.00%	100.00%	1.000 (1.0, 1.0)	0.000
	S	0	4					
LEV	R	14	0	100.00%	100.00%	100.00%	1.000 (1.0, 1.0)	0.000
	S	0	5					
AMX	R	0	4		78.95%	78.95%	0.000 (-0.871, 0.871)	1.000
	S	0	15					
FR	R	0	3		84.21%	84.21%	0.000 (-1.038, 1.038)	1.000
	S	0	16					
TET	R	0	9		52.63%	52.63%	0.000 (-0.474, 0.474)	1.000
	S	0	10					
MTZ	R	18	1	100.00%		94.74%	0.000 (-1.908, 1.908)	1.000
	S	0	0					

In the quinolone resistance-determining region (QRDR) of the *gyrA* gene, mutations at N87 (K, I, T) and D91 (Y, G, H) are hotspots associated with LEV resistance (Chu et al., 2020). These specific mutations impair the ability of LEV to bind to its target, thereby reducing the bactericidal activity of the drug (Ansari and Yamaoka, 2022; Šamanić et al., 2023). Fourteen strains exhibited resistance to LEV and harbored quinolone resistance mutations within the QRDR of *gyrA* (N87I/K, D91N/G). Both phenotypic and genotypic methods showed perfect consistency in detecting LEV resistance (kappa coefficient = 1.000; 95% CI, 1.0 to 1.0) (**Table 6**), indicating high concordance between phenotypic and genotypic assessments in predicting LEV resistance in these isolates.

In this study, among the 18 phenotypically resistant strains, two strains (HN07, HN13) carried nonsense mutations at amino acid positions 35 and 75, resulting in the synthesis of truncated or non-functional proteins. In addition to truncating mutations, amino acid substitutions in *rdxA* were also associated with MTZ resistance (Huang et al., 2024). Of the 18 phenotypically resistant strains, only five (HN03, HN08, HN14, HN15, and HN18) were found to have R16H/C mutations, and two strains (HN02 and HN09) exhibited M21 mutations. The detection of truncated *rdxA* genes or R16 site mutations was fully consistent with the phenotypic resistance results, showing a 100% correlation.

3. *H. pylori* lineage tree analyses.

**Figure 5:**
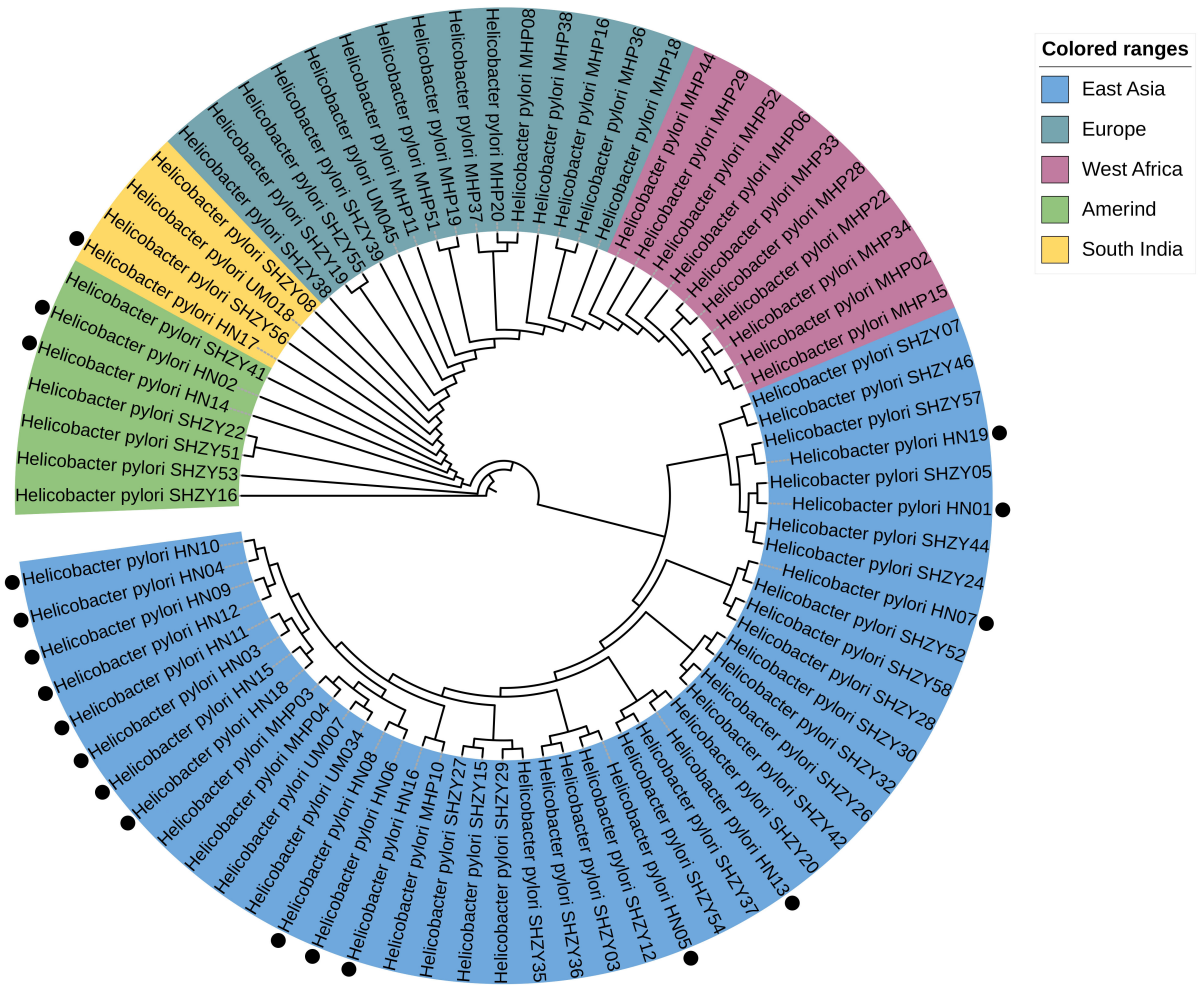
Constructed based on the amino acid and nucleotide sequences of 161 sets of single-copy genes common to 80 Helicobacter pylori genomes. The genomes marked with black dots are the 19 genomes evaluated in our study. These genes were used for multiple sequence alignment, followed by the construction of a phylogenetic tree using the maximum likelihood method.

In this study, the phylogenetic positions of the 19 strains were determined by utilizing WGS data and comparing them with 80 well-classified complete genomes in the database. Subsequently, a phylogenetic tree was constructed. The analysis revealed that 16 strains (84.2% of the total) clustered closely with the East Asian *H. pylori* lineage as expected. Two strains (10.5%), namely HN0_2_ and HN14, were identified to align with the Amerind lineage. Only one strain (5.3%), designated as HN17, was found to be associated with the South Indian lineage. It is noteworthy that none of the genomes exhibited a significant phylogenetic relationship with either the European or West African lineages (**Figure 5**).

## Discussion

In this study, we conducted antibiotic susceptibility testing on 140 *H. pylori* isolates. The findings revealed notable resistance levels to multiple antibiotics, notably MTZ, CLA, and LEV. Of particular concern was the high resistance rate observed for MTZ, reaching 93.6%. According to previous reports, *H. pylori* resistance to MTZ in mainland China is estimated at 70.14% (95% CI: 29.53%-37.46%), while globally, MTZ resistance ranges from 10% to over 90%, depending on geographical location and variations in patient populations with differing patterns of MTZ use (Zeng et al., 2024; Marques et al., 2019; Megraud et al., 2021; Tshibangu-Kabamba and Yamaoka, 2021; Gong et al., 2023). Further examination of MIC values indicated that the sensitivity thresholds for the majority of resistant strains exceeded 32 μg/mL, significantly compromising the efficacy of this antibiotic in the management of *H. pylori* infections. MTZ is considered an alternative treatment option for patients who are allergic to or resistant to CLA or AMX (Gisbert and Pajares, 2005), and it has been widely used in the treatment of anaerobic infections, particularly parasitic infections (Löfmark et al., 2010). However, the emergence of resistance is often closely associated with the historical use of this medication, especially in the context of treating gynecological and dental infections with nitroimidazole compounds, which may exacerbate the further development of resistance (Mégraud, 2004). Moreover, the correlation between MTZ resistance and treatment failure differs significantly from that of CLA resistance (McMahon et al., 2003; Thung et al., 2016; Zhong et al., 2021; Malfertheiner et al., 2022). Due to the diversity of microbiological testing methods and issues related to measurement reproducibility, the reported *in vitro* resistance rates for MTZ often exhibit considerable variability across studies (Mégraud, 2004). However, these discrepancies may also reflect potential trends in the development of resistance. Therefore, further investigation and confirmation of the high incidence of MTZ resistance and its potential consequences are warranted.

Resistance rates to CLA and LEV were recorded at 40.0% and 37.9%, respectively, while the overall resistance rates of *H. pylori* in mainland China to CLA and LEV were 30.72% (95% CI:27.53%-33.99%) and 32.98 (95% CI: 28.73%-37.37%), respectively (Zeng et al., 2024). This indicates a significant decline in the effectiveness of the conventional triple therapy approach for *H. pylori* in the studied region. Furthermore, when considering these resistance rates in a global context, the CLA resistance level in Hainan Province (40.0%) is significantly higher than that observed in other Southeast Asian countries, such as Thailand, Singapore, Malaysia, Indonesia, Cambodia, Japan, and South Korea, where resistance rates range from 12.9% to 35.5% (Chang et al., 2019; Hong et al., 2024; Okimoto et al., 2024). In addition, a study examining H. pylori isolates, it was found that the CLA resistance rate exceeded 15% across all subregions of the United States and European countries. Specifically, in the United States, the highest resistance rate was recorded in the Southeastern region, reaching 24.9% (47/189). Among European countries, the Czech Republic exhibited the highest resistance rate at 26.7% (4/15) (Mégraud et al., 2023). Moreover, a study indicated that, during the European Helicobacter pylori management registry (Hp-EuReg) conducted from 2013 to 2020, the resistance rate to LEV after the first eradication treatment reached 28%. This resistance rate significantly increased to over 45% among patients who underwent more than two eradication attempts (Bujanda et al., 2021). This phenomenon suggests that repeated treatments may contribute to the enhancement of resistance. Therefore, it is crucial to place greater emphasis on the selection of treatment regimens and the rationale for antibiotic usage in clinical practice.

Our investigation delved into the correlation between *H. pylori* genotypes and phenotypic resistance, unveiling a significant alignment. Specifically, resistance to CLA was strongly associated with the presence of the A2143G mutation in the 23S rRNA gene, identified in all CLA-resistant strains (Gong et al., 2020). For LEV, resistance was linked to specific mutations in the *gyrA* gene. The genotyping outcomes demonstrated a high predictive value for resistance to CLA and LEV, with Kappa coefficients of 0.910 and 0.938, respectively. These results underscore the pivotal role of molecular diagnostics in precision medicine, streamlining diagnostic processes and enabling tailored treatment approaches.

A previous prospective study conducted by our team demonstrated that both a 14-day quadruple regimen consisting of a PPI combined with bismuth, AMX, and FR, and a 14-day high-dose dual therapy regimen of PPI and AMX, achieved eradication rates exceeding 90% in Hainan (Zhang et al., 2023). Of the 140 isolates studied, none displayed resistance to AMX, TET, and FR, in stark contrast to a systematic review of antibiotic resistance in *H. pylori* in China, which reported resistance rates of 2.41% (95% CI 1.43%-3.60%),2.53% (95% CI 1.19%-4.28%) and 1.54% (95% CI 0.28%-3.62%) (Zeng et al., 2024; Zhong et al., 2021; Wang et al., 2023; Wang et al., 2024).However, in Gansu Province, located in the northwest region, the resistance rates of Helicobacter pylori to AMX, FR, and TET are notably high, recorded at 12.42%, 20.5%, and 19.25%, respectively. Whether this situation is associated with the excessive use of antibiotics in clinical or agricultural settings in the region requires further investigation (Zhong et al., 2021; Hu et al., 2023). Comparatively, resistance rates in Southeast Asian countries vary from 0.8% to 9.1% for AMX and 2.8% to 5.8% for FR, respectively (Ang et al., 2016; Sukri et al., 2021).

Notably, approximately 25% of the *H. pylori* strains exhibit MDR, defined as resistance to three or more antibiotics. This increased complexity in treatment underscores the need for more sophisticated antibiotic management strategies in clinical settings. The development of MDR is influenced by various factors, including national antibiotic consumption patterns, inappropriate antibiotic utilization, treatment failures, and bacterial characteristics such as genetic mutations, bacterial diversity, as well as host and environmental conditions (Boyanova et al., 2019). Among these factors, the upregulation of multidrug efflux pump systems plays a critical role in MDR acquisition (Ge et al., 2018; Gong et al., 2024). The four primary Resistance-Nodulation-Division (RND) efflux pump families in *H. pylori*—HefABC, HefDEF, HefGHI, and HP1487-HP1489 (Bina et al., 2000; Cai et al., 2020)—reduce the effective concentrations of structurally diverse antibiotics within the bacterial cell (Karbalaei et al., 2022), thereby diminishing their antibacterial effects. Bacteria enhance their defense against antibiotics through genetic mutations or by increasing the expression of efflux pump genes (Elshenawi et al., 2023).

In contrast to PCR and Sanger sequencing methods, WGS emerges as a time- and cost-effective approach for detecting and evaluating antibiotic resistance gene profiles. In our investigation into the application of WGS for identifying molecular markers of resistance in *H. pylori*, comprehensive whole-genome analyses were performed on 19 clinical isolates. The study unveiled a notable correlation between resistance to CLA and LEV and specific genetic mutations, essential for enhancing resistance detection workflows and designing personalized treatment strategies.

Specifically, our findings indicated that 15 CLA-sensitive strains harbored the A2143G point mutation in the 23S rRNA gene, while four LEV-sensitive strains (HN05, HN07, HN11, and HN14) displayed the N87K mutation at the *gyr*A gene locus. The presence of the A2143G mutation was closely associated with elevated MIC values for CLA, thereby augmenting the risk of treatment failure by up to 60%. Furthermore, the interplay of environmental factors and genetic background on phenotypic susceptibility was evident, as observed in three strains exhibiting significant differences in sensitivity or resistance to the two antibiotics.

MTZ, a component of the triple therapy regimen for *H. pylori*, exhibits resistance rates reaching 100% in certain regions, despite the precise mechanisms of resistance remaining incompletely elucidated (Elshenawi et al., 2023). In this study, we identified the R16H/C mutation in the rdxA gene in 5 strains (26.3%, 5/19) that all demonstrated MTZ resistance. The prevalence of the R16H mutation, found in the majority of MTZ-resistant isolates, aligns with previous studies and underscores its pivotal role in resistance development. Interestingly, despite possessing mutations in the rdxA sequence, the HN16 strain exhibited sensitivity to MTZ based on E-test outcomes, suggesting that resistance may stem from multifaceted factors. Future research should delve into alternative potential mechanisms such as efflux pump overexpression or defects in membrane porin proteins.

Our analysis highlights that the primary resistance rate of *H. pylori* to AMX remains low in the Hainan region. Established mechanisms of AMX resistance encompass β-lactamase secretion, structural alterations in pbp1, efflux pump activity, and biofilm formation (Cimuanga-Mukanya et al., 2024; Kwon et al., 2003). However, the direct correlation between these mechanisms and the observed decline in resistance associated with freezing remains ambiguous (Gautier et al., 2013). In China, gastric mucosal samples utilized for *H. pylori* isolation and antibiotic susceptibility testing are typically transported to laboratories in a frozen state (Shu et al., 2018). This process may impact the AMX resistance of certain strains, as approximately 20% to 50% of initially AMX-resistant *H. pylori* strains lose resistance post-freezing, demonstrating a sensitive phenotype instead (Han et al., 2021). This phenomenon could lead to an underestimation of actual AMX resistance rates.

While the A926G mutation in the 16S rRNA gene was detected in six TET-sensitive isolates, further investigation is required to determine its significance in relation to low-level resistance and phenotypic susceptibility. In a recent study, researchers examined the correlation between mutations identified directly from formalin-fixed, paraffin-embedded (FFPE) tissue samples and patient prognosis. Among the six patients harboring the A926G mutation, only one encountered treatment failure. Yet, to comprehensively grasp the clinical implications of this specific mutation, especially among patient cohorts undergoing TET-based treatment protocols, additional large-scale studies are warranted (Nezami et al., 2019; Saranathan et al., 2020).

*H. pylori* can be categorized into seven distinct phylogenetic lineages based on geographic regions (Falush et al., 2003; Kersulyte et al., 2010). The phylogenetic analysis in this study reveals that the six strains originating from a single region do not align with a unified *H. pylori* lineage. In addition to the majority of strains falling under the HpEastAsia lineage, some are distributed among the HspAmerind and South India lineages. This finding mirrors a study conducted in the Ningxia region of China (Zhou et al., 2022), where the HpEastAsia lineage dominates. Model-based studies propose that *H. pylori* originated approximately 58,000 years ago in East Africa and disseminated with human migrations. The bacterium has evolved in tandem with its human hosts over extended periods, along with the HpAsia2 lineage from the Indian subcontinent and Central Asian populations of European descent. Owing to the substantial genomic adaptability of *H. pylori*, disparate lineages may merge over time, complicating efforts to sustain the distinctiveness of *H. pylori* within a stable population. This process partly accounts for the dispersion of strains across diverse lineages. Furthermore, the intensification of globalization may amplify strain diversity.

Our study had several limitations. Firstly, the sample collection was restricted to Hainan Province and drawn from specific medical centers and regions. This geographical limitation may affect the generalizability of our findings to other areas in China or to different countries. Secondly, our samples did not include strains resistant to AMX, TET, and FR. Although the sample size of 140 *H. pylori* isolates is considered sufficient for preliminary analyses, this limitation may hinder the feasibility of more complex multivariable analyses. In particular, the high rate of MTX resistance is concerning; however, given the relatively small number of isolates in this study, the impact of these results is limited. Future studies with larger sample sizes are needed to validate these findings. Additionally, only 19 isolates underwent WGS, which may somewhat constrain our ability to comprehensively interpret specific resistance factors. Finally, our study lacked additional data that could enhance our understanding of resistance-related characteristics, such as patients’ clinical symptoms and prior antibiotic usage records. Such information would aid in elucidating the relationship between genotypes and resistance mechanisms. Nevertheless, we established multiple sub-centers in Hainan Province to conduct dynamic research on *H. pylori* infections, treatment, and antibiotic resistance testing. The establishment of these sub-centers lays a solid foundation for future in-depth exploration of this field and provides strong support for larger-scale studies. Such efforts will help confirm our current findings and promote improvements in related clinical practices.

Overall, given the high global prevalence of *H. pylori* infections and the growing issue of antibiotic resistance, there is an urgent need to develop new effective prevention and treatment strategies. In the absence of an effective vaccine, eradication therapy remains the primary option. The data from this study are crucial for formulating effective treatment protocols aimed at eradicating *H. pylori* in Hainan Province and advancing personalized medicine.

## Data Availability

The datasets presented in this study can be found in online repositories. The names of the repository/repositories and accession number(s) can be found below: https://www.ncbi.nlm.nih.gov/, PRJNA1171892.

## References

[B1] Ang T. L. Fock K. M. Ang D. Kwek A. B. E. Teo E. K. Dhamodaran S. (2016). The changing profile of Helicobacter pylori antibiotic resistance in Singapore: a 15-year study. Helicobacter 21, 261–265. doi: 10.1111/hel.2016.21.issue-4 26774006

[B2] Ansari S. Yamaoka Y. (2022). Helicobacter pylori infection, its laboratory diagnosis, and antimicrobial resistance: a perspective of clinical relevance. Clin. Microbiol. Rev. 35, e00258–e00221. doi: 10.1128/cmr.00258-21 35404105 PMC9491184

[B3] Bauer B. Meyer T. F. (2011). The human gastric pathogen Helicobacter pylori and its association with gastric cancer and ulcer disease. Ulcers 2011, 340157. doi: 10.1155/2011/340157

[B4] Bina J. E. Alm R. A. Uria-Nickelsen M. Thomas S. R. Trust T. J. Hancock R. E. W. (2000). Helicobacter pylori uptake and efflux: basis for intrinsic susceptibility to antibiotics. vitro. Antimicrobial Agents chemotherapy 44, 248–254. doi: 10.1128/AAC.44.2.248-254.2000 10639345 PMC89666

[B5] Boyanova L. Hadzhiyski P. Kandilarov N. Markovska R. Mitov I. (2019). Multidrug resistance in Helicobacter pylori: current state and future directions. Expert Rev. Clin. Pharmacol. 12, 909–915. doi: 10.1080/17512433.2019.1654858 31424296

[B6] Bujanda L. Nyssen O. P. Vaira D. Saracino I. M. Fiorini G. Lerang F.. (2021). Antibiotic Resistance Prevalence and Trends in Patients Infected with Helicobacter pylori in the Period 2013-2020: Results of the European Registry on H. pylori Management (Hp-EuReg). Antibiotics (Basel Switzerland) 10 (9). doi: 10.3390/antibiotics10091058 PMC8471667 34572640

[B7] Cai Y. Wang C. Chen Z. Xu Z. Li H. Li W.. (2020). Transporters HP0939, HP0497, and HP0471 participate in intrinsic multidrug resistance and biofilm formation in Helicobacter pylori by enhancing drug efflux. Helicobacter 25, e12715. doi: 10.1111/hel.12715 32548895

[B8] Chang Y. W. Ko W. J. Oh C. H. Park Y. M. Oh S. J. Moon J. R.. (2019). Clarithromycin resistance and female gender affect Helicobacter pylori eradication failure in chronic gastritis. Korean J. Internal Med. 34, 1022. doi: 10.3904/kjim.2018.054 29898576 PMC6718756

[B9] Chen S. Shen W. Liu Y. Dong Q. Shi Y. (2023). Efficacy and safety of triple therapy containing berberine, amoxicillin, and vonoprazan for Helicobacter pylori initial treatment: A randomized controlled trial. Chin. Med. J. 136, 1690–1698. doi: 10.1097/CM9.0000000000002696 37469024 PMC10344537

[B10] Chu A. Wang D. Guo Q. Lv Z. Yuan Y. Gong Y. (2020). Molecular detection of H. pylori antibiotic-resistant genes and molecular docking analysis. FASEB J. 34, 610–618. doi: 10.1096/fj.201900774R 31914672

[B11] Cimuanga-Mukanya A. Tshibangu-Kabamba E. Kisoko P. Fauzia K. A. Tshibangu F. M. Wola A. T.. (2024). Synergistic effects of novel penicillin-binding protein 1A amino acid substitutions contribute to high-level amoxicillin resistance of Helicobacter pylori. mSphere, e00089–e00024. doi: 10.1128/msphere.00089-24 39087788 PMC11351044

[B12] Crowe S. E. (2019). Helicobacter pylori infection. New Engl. J. Med. 380, 1158–1165. doi: 10.1056/NEJMcp1710945 30893536

[B13] Elshenawi Y. Hu S. Hathroubi S. (2023). Biofilm of Helicobacter pylori: Life cycle, features, and treatment options. Antibiotics 12, 1260. doi: 10.3390/antibiotics12081260 37627679 PMC10451559

[B14] Falush D. Wirth T. Linz B. Pritchard J. K. Stephens M. Kidd M.. (2003). Traces of human migrations in Helicobacter pylori populations. science 299, 1582–1585. doi: 10.1126/science.1080857 12624269

[B15] Fauzia K. A. Alfaray R. I. Yamaoka Y. (2023). Advantages of whole genome sequencing in mitigating the Helicobacter pylori antimicrobial resistance problem. Microorganisms. 11 (5), 1239. doi: 10.3390/microorganisms11051239 37317213 PMC10220574

[B16] Fazal F. (2019). European Committee on Antimicrobial Susceptibility Testing and Clinical and Laboratory Standards Institute breakpoints—the only point that matters in candidemia? J. Thorac. Dis. 11, S1412. doi: 10.21037/jtd.2019.03.12 31245147 PMC6560619

[B17] Fukase K. Kato M. Kikuchi S. Inoue K. Uemura N. Okamoto S.. (2008). Effect of eradication of Helicobacter pylori on incidence of metachronous gastric carcinoma after endoscopic resection of early gastric cancer: an open-label, randomised controlled trial. Lancet 372, 392–397. doi: 10.1016/S0140-6736(08)61159-9 18675689

[B18] Gautier J. Passot S. Pénicaud C. Guillemin H. Cenard S. Lieben P.. (2013). A low membrane lipid phase transition temperature is associated with a high cryotolerance of Lactobacillus delbrueckii subspecies bulgaricus CFL1. J. dairy Sci. 96, 5591–5602. doi: 10.3168/jds.2013-6802 23810590

[B19] Ge X. Cai Y. Chen Z. Gao S. Geng X. Li Y.. (2018). Bifunctional enzyme SpoT is involved in biofilm formation of Helicobacter pylori with multidrug resistance by upregulating efflux pump Hp1174 (gluP). Antimicrobial Agents chemotherapy 62, 10–1128. doi: 10.1128/AAC.00957-18 PMC6201075 30181372

[B20] Gisbert J. P. Pajares J. M. (2005). Helicobacter pylori "rescue" therapy after failure of two eradication treatments. Helicobacter 10, 363–372. doi: 10.1111/j.1523-5378.2005.00324.x 16181345

[B21] Goh K. L. Navaratnam P. (2011). High Helicobacter pylori resistance to metronidazole but zero or low resistance to clarithromycin, levofloxacin, and other antibiotics in Malaysia. Helicobacter 16, 241–245. doi: 10.1111/j.1523-5378.2011.00841.x 21585611

[B22] Gong E. J. Ahn J. Y. Kim J. M. Lee S. M. Na H. K. Lee J. H.. (2020). Genotypic and phenotypic resistance to clarithromycin in Helicobacter pylori strains. J. Clin. Med. 9, 1930. doi: 10.3390/jcm9061930 32575584 PMC7356929

[B23] Gong X. Wang Y. An Y. Li Z. Liu D. Yong X. (2024). The crosstalk between efflux pump and resistance gene mutation in Helicobacter pylori. Gut Microbes 16, 2379439. doi: 10.1080/19490976.2024.2379439 39052777 PMC11275522

[B24] Gong Y. Yuan Y. (2018). Resistance mechanisms of Helicobacter pylori and its dual target precise therapy. Crit. Rev. Microbiol. 44, 371–392. doi: 10.1080/1040841X.2017.1418285 29293032

[B25] Gong Y. Zhai K. Sun L. He L. Wang H. Guo Y.. (2023). RdxA diversity and mutations associated with metronidazole resistance of Helicobacter pylori. Microbiol. Spectr. 11, e03903–e03922. doi: 10.1128/spectrum.03903-22 36943041 PMC10100817

[B26] Gutiérrez E. G. Maldonado J. E. Castellanos-Morales G. Eguiarte L. E. Martínez-Méndez N. Ortega J. (2024). Unraveling genomic features and phylogenomics through the analysis of three Mexican endemic Myotis genomes. PeerJ 12, e17651. doi: 10.7717/peerj.17651 38993980 PMC11238727

[B27] Han X. Zhang Y. He L. Fan R. Sun L. Fan D.. (2021). Genetic and transcriptomic variations for amoxicillin resistance in Helicobacter pylori under cryopreservation. Pathogens 10, 676. doi: 10.3390/pathogens10060676 34070823 PMC8229390

[B28] Hanafiah A. Abd Aziz S. N. A. Nesran Z. N. M. Wezen X. C. Ahmad M. F. (2024). Molecular investigation of antimicrobial peptides against Helicobacter pylori proteins using a peptide-protein docking approach. Heliyon 10 (6). doi: 10.1016/j.heliyon.2024.e28128 PMC10963377 38533069

[B29] Hansomburana P. Anantapanpong S. Sirinthornpunya S. Chuengyong K. Rojborwonwittaya J. (2012). Prevalence of single nucleotide mutation in clarithromycin resistant gene of Helicobacter pylori: a 32-months prospective study by using hybridization real time polymerase chain reaction. J. Med. Assoc. Thai 95, S28–S35. 22619884

[B30] Hong T.-C. El-Omar E. M. Kuo Y.-T. Wu J.-Y. Chen M.-J. Chen C.-C.. (2024). Primary antibiotic resistance of Helicobacter pylori in the Asia-Pacific region between 1990 and 2022: an updated systematic review and meta-analysis. Lancet Gastroenterol. Hepatol. 9, 56–67. doi: 10.1016/S2468-1253(23)00281-9 37972625

[B31] Hooi J. K. Y. Lai W. Y. Ng W. K. Suen M. M. Y. Underwood F. E. Tanyingoh D.. (2017). Global prevalence of Helicobacter pylori infection: systematic review and meta-analysis. Gastroenterology 153, 420–429. doi: 10.1053/j.gastro.2017.04.022 28456631

[B32] Hu S. Zhou Y. Deng Y. Bo Y. Chen X. Yang W.. (2023). Characteristics of phenotypic antibiotic resistance of Helicobacter pylori and its correlation with genotypic antibiotic resistance: A retrospective study in Ningxia. Helicobacter 28, e12960. doi: 10.1111/hel.12960 37042045

[B33] Huang J. Li Z. Ge F. Sun C. Deng Z. Yao W.. (2024). Functional determination of site-mutations in rdxA involved in metronidazole resistance of Helicobacter pylori. Front. Cell Dev. Biol. 12, 1435064. doi: 10.3389/fcell.2024.1435064 39100097 PMC11294100

[B34] Karbalaei M. Keikha M. Abadi A. T. B. (2022). Prevalence of primary multidrug-resistant Helicobacter pylori in children: a systematic review and meta-analysis. Arch. Med. Res. 53, 634–640. doi: 10.1016/j.arcmed.2022.08.010 36089418

[B35] Kersulyte D. Kalia A. Gilman R. H. Mendez M. Herrera P. Cabrera L.. (2010). Helicobacter pylori from Peruvian amerIndians: traces of human migrations in strains from remote Amazon, and genome sequence of an Amerind strain. PloS One 5, e15076. doi: 10.1371/journal.pone.0015076 21124785 PMC2993954

[B36] Kumar S. Stecher G. Li M. Knyaz C. Tamura K. (2018). MEGA X: molecular evolutionary genetics analysis across computing platforms. Mol. Biol. Evol. 35, 1547–1549. doi: 10.1093/molbev/msy096 29722887 PMC5967553

[B37] Kwon D. H. Dore M. P. Kim J. J. Kato M. Lee M. Wu J. Y.. (2003). High-level β-lactam resistance associated with acquired multidrug resistance in Helicobacter pylori. Antimicrobial Agents chemotherapy 47, 2169–2178. doi: 10.1128/AAC.47.7.2169-2178.2003 12821464 PMC161855

[B38] Liu L. Shi H. Shi Y. Wang A. Guo N. Li F.. (2024). Vonoprazan-based therapies versus PPI-based therapies in patients with H. pylori infection: Systematic review and meta-analyses of randomized controlled trials. Helicobacter 29, e13094. doi: 10.1111/hel.13094 38790090

[B39] Löfmark S. Edlund C. Nord C. E. (2010). Metronidazole is still the drug of choice for treatment of anaerobic infections. Clin. Infect. diseases: an Off. Publ. Infect. Dis. Soc. America 50 Suppl 1, S16–S23. doi: 10.1086/647939 20067388

[B40] Lyu T. Cheung K. S. Deng Z. Ni L. Chen C. Wu J.. (2023). Whole genome sequencing reveals novel genetic mutations of Helicobacter pylori associating with resistance to clarithromycin and levofloxacin. Helicobacter 28, e12972. doi: 10.1111/hel.12972 36965192

[B41] Malfertheiner P. Camargo M. C. El-Omar E. Liou J.-M. Peek R. Schulz C.. (2023). Helicobacter pylori infection. Nat. Rev. Dis. Primers 9, 19. doi: 10.1038/s41572-023-00431-8 37081005 PMC11558793

[B42] Malfertheiner P. Megraud F. Rokkas T. Gisbert J. P. Liou J. M. Schulz C.. (2022). Management of Helicobacter pylori infection: the Maastricht VI/Florence consensus report. Gut. doi: 10.1136/gutjnl-2022-327745 35944925

[B43] Mannion A. Dzink-Fox J. Shen Z. Piazuelo M. B. Wilson K. T. Correa P.. (2021). Helicobacter pylori antimicrobial resistance and gene variants in high-and low-gastric-cancer-risk populations. J. Clin. Microbiol. 59, 10–1128. doi: 10.1128/JCM.03203-20 PMC8091839 33692136

[B44] Marques B. Donato M. M. Cardoso O. Luxo C. Martinho A. Almeida N. (2019). Study of rdxA and frxA genes mutations in metronidazole-resistant and-susceptible Helicobacter pylori clinical isolates from the central region of Portugal. J. Global antimicrobial resistance 17, 300–304. doi: 10.1016/j.jgar.2019.01.008 30658199

[B45] Marques A. T. Vítor J. M. B. Santos A. Oleastro M. Vale F. F. (2020). Trends in Helicobacter pylori resistance to clarithromycin: from phenotypic to genomic approaches. Microbial Genomics 6, e000344. doi: 10.1099/mgen.0.000344 32118532 PMC7200067

[B46] McColl K. E. L. (2010). Helicobacter pylori infection. New Engl. J. Med. 362, 1597–1604. doi: 10.1056/NEJMcp1001110 20427808

[B47] McMahon B. J. Hennessy T. W. Bensler J. M. Bruden D. L. Parkinson A. J. Morris J. M.. (2003). et al: The relationship among previous antimicrobial use, antimicrobial resistance, and treatment outcomes for Helicobacter pylori infections. Ann. Internal Med. 139, 463–469. doi: 10.7326/0003-4819-139-6-200309160-00008 13679322

[B48] Mégraud F. (2004). H pylori antibiotic resistance: prevalence, importance, and advances in testing. Gut 53, 1374–1384. doi: 10.1136/gut.2003.022111 15306603 PMC1774187

[B49] Megraud F. Bruyndonckx R. Coenen S. Wittkop L. Huang T.-D. Hoebeke M.. (2021). Helicobacter pylori resistance to antibiotics in Europe in 2018 and its relationship to antibiotic consumption in the community. Gut 70, 1815–1822. doi: 10.1136/gutjnl-2021-324032 33837118

[B50] Mégraud F. Graham D. Y. Howden C. W. Trevino E. Weissfeld A. Hunt B.. (2023). Rates of antimicrobial resistance in helicobacter pylori isolates from clinical trial patients across the US and Europe. Am. J. Gastroenterol. 118, 269–275. doi: 10.14309/ajg.0000000000002045 36191284 PMC9889195

[B51] Nezami B. G. Jani M. Alouani D. Rhoads D. D. Sadri N. (2019). Helicobacter pylori mutations detected by next-generation sequencing in formalin-fixed, paraffin-embedded gastric biopsy specimens are associated with treatment failure. J. Clin. Microbiol. 57, 10–1128. doi: 10.1128/JCM.01834-18 PMC6595463 31068413

[B52] Okimoto T. Ando T. Sasaki M. Ono S. Kobayashi I. Shibayama K.. (2024). Antimicrobial-resistant Helicobacter pylori in Japan: Report of nationwide surveillance for 2018–2020. Helicobacter 29, e13028. doi: 10.1111/hel.13028 37823466

[B53] Okubo H. Akiyama J. Kobayakawa M. Kawazoe M. Mishima S. Takasaki Y.. (2020). Vonoprazan-based triple therapy is effective for Helicobacter pylori eradication irrespective of clarithromycin susceptibility. J. Gastroenterol. 55, 1054–1061. doi: 10.1007/s00535-020-01723-6 32930864

[B54] Qian H.-S. Li W.-J. Dang Y.-N. Li L.-R. Xu X.-B. Yuan L.. (2023). Ten-day vonoprazan-amoxicillin dual therapy as a first-line treatment of Helicobacter pylori infection compared with bismuth-containing quadruple therapy. Off. J. Am. Coll. Gastroenterology| ACG 118, 627–634. doi: 10.14309/ajg.0000000000002086 36729890

[B55] Rimbara E. Noguchi N. Kawai T. Sasatsu M. (2008). Mutations in penicillin-binding proteins 1, 2 and 3 are responsible for amoxicillin resistance in Helicobacter pylori. J. antimicrobial chemotherapy 61, 995–998. doi: 10.1093/jac/dkn051 18276599

[B56] Rokkas T. Ekmektzoglou K. (2023). Advances in the pharmacological and regulatory management of multidrug resistant Helicobacter pylori. Expert Rev. Clin. Pharmacol. 16, 1229–1237. doi: 10.1080/17512433.2023.2282061 37937850

[B57] Rosli N. A. Al-Maleki A. R. Loke M. F. Chua E. G. Alhoot M. A. Vadivelu J. (2023). Polymorphism of virulence genes and biofilm associated with in *vitro* induced resistance to clarithromycin in Helicobacter pylori. Gut Pathog. 15, 52. doi: 10.1186/s13099-023-00579-4 37898785 PMC10613384

[B58] Šamanić I. Dadić B. Sanader Maršić Ž. Dželalija M. Maravić A. Kalinić H.. (2023). Molecular characterization and mutational analysis of clarithromycin-and levofloxacin-resistance genes in helicobacter pylori from gastric biopsies in Southern Croatia. Int. J. Mol. Sci. 24, 14560. doi: 10.3390/ijms241914560 37834008 PMC10572715

[B59] Saranathan R. Levi M. H. Wattam A. R. Malek A. Asare E. Behin D. S.. (2020). Helicobacter pylori infections in the Bronx, New York: surveying antibiotic susceptibility and strain lineage by whole-genome sequencing. J. Clin. Microbiol. 58, 10–1128. doi: 10.1128/JCM.01591-19 PMC7041580 31801839

[B60] Shu X. Yin G. Liu M. Peng K. Zhao H. Jiang M. (2018). Antibiotics resistance of Helicobacter pylori in children with upper gastrointestinal symptoms in Hangzhou, China. Helicobacter 23, e12481. doi: 10.1111/hel.2018.23.issue-3 29528162

[B61] Sukri A. Hanafiah A. Yusoff H. Shamsul Nizam N. A. Nameyrra Z. Wong Z.. (2022). Multidrug-resistant Helicobacter pylori strains: a five-year surveillance study and its genome characteristics. Antibiotics 11, 1391. doi: 10.3390/antibiotics11101391 36290049 PMC9598532

[B62] Sukri A. Lopes B. S. Hanafiah A. (2021). The emergence of multidrug-resistant Helicobacter pylori in Southeast Asia: A systematic review on the trends and intervention strategies using antimicrobial peptides. Antibiotics 10, 1061. doi: 10.3390/antibiotics10091061 34572643 PMC8465560

[B63] Tacconelli E. Carrara E. Savoldi A. Harbarth S. Mendelson M. Monnet D. L.. (2018). Discovery, research, and development of new antibiotics: the WHO priority list of antibiotic-resistant bacteria and tuberculosis. Lancet Infect. Dis. 18, 318–327. doi: 10.1016/S1473-3099(17)30753-3 29276051

[B64] Thung I. Aramin H. Vavinskaya V. Gupta S. Park J. Y. Crowe S. E.. (2016). Review article: the global emergence of Helicobacter pylori antibiotic resistance. Alimentary Pharmacol. Ther. 43, 514–533. doi: 10.1111/apt.2016.43.issue-4 PMC5064663 26694080

[B65] Tshibangu-Kabamba E. Yamaoka Y. (2021). Helicobacter pylori infection and antibiotic resistance—from biology to clinical implications. Nat. Rev. Gastroenterol. Hepatol. 18, 613–629. doi: 10.1038/s41575-021-00449-x 34002081

[B66] Vakil N. Megraud F. (2007). Eradication therapy for Helicobacter pylori. Gastroenterology 133, 985–1001. doi: 10.1053/j.gastro.2007.07.008 17854602

[B67] Wang Y. Du J. Zhang D. Jin C. Chen J. Wang Z.. (2023). Primary antibiotic resistance in Helicobacter pylori in China: a systematic review and meta-analysis. J. Global Antimicrobial Resistance 34, 30–38. doi: 10.1016/j.jgar.2023.05.014 37315738

[B68] Wang L. Li Z. Tay C. Y. Marshall B. J. Gu B. Tian Y.. (2024). Multicentre, cross-sectional surveillance of Helicobacter pylori prevalence and antibiotic resistance to clarithromycin and levofloxacin in urban China using the string test coupled with quantitative PCR. Lancet Microbe. doi: 10.1016/S2666-5247(24)00027-2 38437848

[B69] Wang B. Lv Z.-F. Wang Y.-H. Wang H. Liu X.-Q. Xie Y.. (2014). Standard triple therapy for Helicobacter pylori infection in China: a meta-analysis. World J. Gastroenterology: WJG 20, 14973. doi: 10.3748/wjg.v20.i40.14973 25356059 PMC4209562

[B70] Windham I. H. Merrell D. S. (2022). Interplay between amoxicillin resistance and osmotic stress in Helicobacter pylori. J. Bacteriology 204, e00045–e00022. doi: 10.1128/jb.00045-22 PMC9112972 35389254

[B71] Xiong M. Mohammed Aljaberi H. S. Khalid Ansari N. Sun Y. Yin S. Nasifu L.. (2023). Phenotype and genotype analysis for Helicobacter pylori antibiotic resistance in outpatients: A retrospective study. Microbiol. Spectr. 11, e00550–e00523. doi: 10.1128/spectrum.00550-23 37732751 PMC10580949

[B72] Zeng S. Kong Q. Wu X. Duan M. Nan X. Yang X.. (2024). Antibiotic Resistance of Helicobacter pylori in Mainland China: A Focus on Geographic Differences through Systematic Review and Meta-analysis. Int. J. Antimicrob. Agents, 107325. 39245326 10.1016/j.ijantimicag.2024.107325

[B73] Zhang S. Wang X. Wise M. J. He Y. Chen H. Liu A.. (2020). Mutations of Helicobacter pylori RdxA are mainly related to the phylogenetic origin of the strain and not to metronidazole resistance. J. Antimicrobial Chemotherapy 75, 3152–3155. doi: 10.1093/jac/dkaa302 32676634

[B74] Zhang X. D. Zhang D. Y. Chen R. X. Chen S. J. Chen C. Zeng F.. (2023). Ilaprazole-amoxicillin dual therapy at high dose as a first-line treatment for helicobacter pylori infection in Hainan: a single-center, open-label, noninferiority, randomized controlled trial. BMC Gastroenterol. 23, 249. doi: 10.1186/s12876-023-02890-5 37488516 PMC10364389

[B75] Zhao L.-J. Huang Y.-Q. Chen B.-P. Mo X.-Q. Huang Z.-S. Huang X.-F.. (2014). Helicobacter pylori isolates from ethnic minority patients in Guangxi: Resistance rates, mechanisms, and genotype. World J. Gastroenterology: WJG 20, 4761. doi: 10.3748/wjg.v20.i16.4761 PMC4000514 24782630

[B76] Zhong Z. Zhang Z. Wang J. Hu Y. Mi Y. He B.. (2021). A retrospective study of the antibiotic-resistant phenotypes and genotypes of Helicobacter pylori strains in China. Am. J. Cancer Res. 11, 5027–5037. 34765309 PMC8569369

[B77] Zhou L. Lu H. Song Z. Lyu B. Chen Y. Wang J.. (2022). 2022 Chinese national clinical practice guideline on Helicobacter pylori eradication treatment. Chin. Med. J. 135, 2899–2910. 36579940 10.1097/CM9.0000000000002546 PMC10106216

[B78] Zhou Y. Zhong Z. Hu S. Wang J. Deng Y. Li X.. (2022). A survey of helicobacter pylori antibiotic-resistant genotypes and strain lineages by whole-genome sequencing in China. Antimicrobial Agents chemotherapy 66, e02188–e02121. doi: 10.1128/aac.02188-21 35652644 PMC9211431

